# Perception of antimicrobial stewardship interventions in Swiss primary care: a mixed-methods survey

**DOI:** 10.3399/BJGPO.2024.0110

**Published:** 2025-04-24

**Authors:** Simeon Schaad, Jelena Dunaiceva, Arnaud Peytremann, Sophie Gendolla, Lauren Clack, Catherine Plüss-Suard, Anne Niquille, Anna Nicolet, Joachim Marti, Noémie Boillat-Blanco, Aline Wolfensberger, Yolanda Mueller

**Affiliations:** 1 Unisanté, University Center for Primary Care and Public Health, Department of Family Medicine, University of Lausanne, Lausanne, Switzerland; 2 Institute for Implementation Science in Health Care, University of Zurich Faculty of Medicine, Zurich, Switzerland; 3 Division of Infectious Diseases and Hospital Epidemiology, University Hospital Zurich, Zurich, Switzerland; 4 Swiss Centre for Antibiotic Resistance (ANRESIS), Institute for Infectious Diseases, University of Bern, Bern, Switzerland; 5 Unisanté, University Center for Primary Care and Public Health, Department of Ambulatory Care, University of Lausanne, Lausanne, Switzerland; 6 Department of Epidemiology and Health Systems, Unisanté, University Center for Primary Care and Public Health, Lausanne, Switzerland; 7 Infectious Diseases Service, Department of Medicine, University Hospital and University of Lausanne, Lausanne, Switzerland

**Keywords:** Antimicrobial stewardship, primary care, implementation

## Abstract

**Background:**

With most antibiotic prescriptions occurring in primary care, antimicrobial stewardship (AMS) interventions must be known, welcomed, and used by primary care physicians (PCPs).

**Aim:**

The main objective of this study was to evaluate the present awareness about, use of, and perceived acceptability, appropriateness, and feasibility of a broad range of interventions.

**Design & setting:**

A cross-sectional survey was distributed to Swiss PCPs from December 2023 to February 2024.

**Method:**

The survey focused on eight AMS interventions: shared decision-making tools, factsheets for physicians, Swiss Federal Office of Public Health (FOPH) information material, national antibiotic guidelines website, audit and feedback, communication skills training, as well as the use of point-of-care C-reactive protein (POC-CRP) and procalcitonin (POC-PCT) to guide prescription. PCPs’ perceived acceptability, appropriateness, and feasibility were assessed using 5-point Likert scales. General expectations regarding AMS were evaluated via qualitative analysis of free-text answers.

**Results:**

Out of 7456 potentially eligible primary care physicians, 355 PCPs answered at least one question (response rate 4.7%). PCPs were most aware of biomarkers to guide antibiotic prescription in respiratory tract infections (RTIs), such as POC-PCT (67.6%) and POC-CRP (61.1%); the FOPH awareness campaign (57.3%); and the national guidelines website (52.7%). All interventions were rated as acceptable, appropriate, and feasible, with respective mean scores out of five of 3.89, 3.91, and 3.81.

**Conclusion:**

Despite the high perceived acceptability, appropriateness, and feasibility of AMS interventions available for RTIs, their real-life impact may be hindered by insufficient awareness. Additional promotion of those tools could increase their uptake by physicians.

## How this fits in

Antimicrobial stewardship (AMS) interventions that are effective in clinical trials sometimes fail to have real-life effect in primary care. Several barriers can jeopardise the implementation of AMS interventions in the outpatient setting. The perspectives of primary care physicians have only been partially explored for the design of most of AMS interventions. Assessing implementation outcomes among primary care physicians may increase understanding of how specific AMS interventions and their implementations are perceived in the outpatient care setting.

## Introduction

Antimicrobial resistance is a global threat with over 30 000 attributable deaths and 800 000 disability-adjusted life years (DALYs) in Europe.^1,2^ With rising resistance rates, driven by the inappropriate use of antimicrobials,^3,4^ the need for AMS, defined as a coherent set of actions that promote using antimicrobials responsibly,^5^ has become evident. Accounting for nearly 90% of all antibiotic prescriptions in Switzerland,^6^ outpatient care is a prime target to implement AMS interventions.^7^ AMS interventions targeting outpatient care can embody several forms,^8^ including educational interventions,^9^ clinical guidelines on antimicrobial treatment,^10^ communication skills training programs,^11^ resistance rate data visualisation,^12^ decision support tools,^13^ diagnostic imaging,^14^ financial incentives,^15^ use of biomarkers to guide prescribing,^16^ antibiotic prescription audit and feedback,^17^ or regulatory measures.^18^ However, several barriers may jeopardise the implementation of AMS interventions in primary care,^19–21^ indicating the need to tailor AMS interventions according to the professional and socio-cultural context.^22^ For example, the GRACE/INTRO study,^23^ which aimed to develop and assess two AMS interventions (communications skills training and point-of-care C-reactive protein [POC-CRP]), showed a reduction of antibiotic prescription in the study context, but its real-word impact was unsuccessful in a nationwide implementation study,^20^ owing to a low uptake of the online training course.

In the pursuit of tailoring AMS interventions to the local needs, assessing implementation outcomes may increase understanding of how specific AMS interventions and their implementation are perceived in the outpatient care setting. Proctor and colleagues defined eight implementation outcomes, including acceptability, appropriateness, and feasibility.^24^ Based on several studies,^24–27^ acceptability, appropriateness, and feasibility are considered ‘leading indicators‘ for implementation success (for example, an increase in acceptability could be followed by an increase in penetration).

In Switzerland, the Swiss Strategy on Antibiotic Resistance (StAR) nationally coordinates AMS interventions in the human, animal, and environmental field using a One Health approach.^28^ StAR promotes several AMS interventions, such as guidelines, shared decision-making tools, resistance rate data representation, consumption data representation, and patient information sheets regarding antibiotic prescriptions. In the Swiss context, primary care physicians (PCPs) include general internal medicine specialists, practicing physicians, and pediatricians working in the ambulatory context, in private practice, or larger outpatient clinics. These physicians are the main prescribers of antibiotics in the outpatient setting and thus a prime target for AMS interventions. PCPs’ perspectives have been partially explored during the development of some of these tools,^29^ but — to our knowledge — not all of them. Additionally, new AMS interventions have since gained popularity, with the recent availability of point-of-care procalcitonin (POC-PCT) that could help PCPs to better differentiate between viral and bacterial infections, and help to identify patients that would benefit the most from an antibiotic treatment.^14,16^ As the implementation of those tests in primary care daily practice is also highly conditional on individual factors, such as PCPs’ own perceptions of these tests,^30–32^ and given the scarcity of literature on the factors impacting the implementation of AMS in primary care,^33^ research on PCPs’ views and attitudes towards AMS interventions is needed for the Swiss context.

Assessing PCPs’ perspectives on AMS interventions is essential since they are one of the main healthcare providers targeted by AMS in Switzerland. Furthermore, AMS interventions targeting RTIs specifically are crucial given the high rate of inappropriate antibiotic prescriptions for this indication, with studies indicating rates as high as 50%.^34^ This study aims 1) to explore which sources of information about antibiotic use are used by Swiss PCPs, either to retrieve information, or to update their knowledge about appropriate antibiotic prescription, 2) to assess the awareness about, as well as the perceived acceptability, appropriateness, and feasibility of several AMS interventions aiming to optimise the prescription of antibiotics in RTIs, and 3) to explore PCPs’ expectations regarding an AMS intervention efficiently supporting them in prescribing antimicrobials more appropriately. Reporting of this study was made according to the checklist for reporting of survey studies (CROSS,^35^).

## Method

### Study design and setting

This online, mixed-methods, cross-sectional survey was conducted from 6 December 2023 to 29 February 2024.

### Data collection

The survey was available in German, French, Italian, and English. The survey contained five parts. Part one focused on socio-demographic characteristics of survey responders. Part two asked PCPs to select and rank 16 information sources about antibiotic prescription considered as AMS interventions (see Supplementary Table 1), to explore PCPs’ preferences for updating their knowledge on appropriate antibiotic prescription. Part three assessed the six general AMS interventions that can be used to guide prescription in RTIs; that is, shared decision-making tools, factsheets for physicians, national guidelines, FOPH patient information material, antibiotic prescription audit and feedback, and communication skills training, as well as of two biomarker-based interventions specific for RTIs: point-of-care C-reactive protein (POC-CRP) and procalcitonin (POC-PCT) (see Supplementary Table 2). To minimise the completion time of the survey, only two of six general AMS interventions, and one out of two biomarker-based AMS interventions were randomly allocated to each participant. As the shared decision-making tool and the factsheets were designed to be used jointly during a given consultation, the two were considered as one AMS intervention to be evaluated together. The allocated AMS interventions were briefly described or shown in the survey, and PCP’s awareness of the existence of the intervention was assessed. Then, PCPs’ perceived acceptability, appropriateness, and feasibility regarding those same AMS interventions were evaluated using 5-point Likert scales, assessing agreement with Proctor’s definitions of each implementation outcomes.^24,36^ Free-text fields were provided for each intervention, allowing PCPs to express any comments they had regarding the evaluated AMS interventions. Some contextual questions were included to evaluate PCPs’ opinions on the design of specific AMS interventions. These questions included PCPs’ preferences regarding the design of an antibiotic prescription audit and feedback intervention, suggestions for new guidelines, and their perception of the time it takes to perform a given AMS intervention. Part four assessed PCPs’ current practices in terms of laboratory tests performed at their practices. Part five assessed PCPs’ expectations about an AMS intervention in Switzerland. The total number of questions varied from 38 to 45, depending on the interventions randomly allocated for evaluation to each PCP, as well as on the aforementioned contextual questions. The estimated completion time was 15 minutes. The full questionnaire is available in the Supplementary material.

### Pretesting

The survey was pre-tested in French among five French-speaking PCPs fulfilling inclusion criteria for the study, selected purposively to represent different age and gender categories. They gave feedback on the wording of the questions, the ease of use of the survey interface, and the time take to complete.

### Target population

The survey targeted Swiss PCPs, defined as holders of a specialist title in general internal medicine^37^ or of a practicing physician certification,^38^ and working in the outpatient sector. This corresponded to 7456 individuals according to statistics of the Swiss Medical Association (FMH) 2022.^39^ Pediatricians were excluded from the study because some AMS interventions evaluated in the survey were designed specifically for adult patients. Similarly, specialists in general internal medicine or practicing physicians who primarily treat children (which is rare in Switzerland but possible) were also excluded. To take part in the study, the Swiss PCPs needed to have a mainly adult (≥80% of patients being ≥16 years old) outpatient population, in either a private practice, a walk-in clinic, or an academic outpatient centre. For physicians with multiple FMH specialist titles (for example, infectious diseases specialist, tropical and travel medicine specialist), they had to consider family medicine as their main clinical activity. The participation to a previous AMS trial was not an exclusion criterion. These inclusion criteria were assessed at the beginning of the survey, and the participants were excluded from the survey if they were not eligible based on their responses.

### Sampling techniques, sample size, and representativeness of the sample

Physicians were self-selected to take part in the study. To be able to estimate proportions of answers for the implementation outcomes of 30% with a precision of 10%, we aimed at a sample size of 81 PCPs per evaluated intervention. We assessed representativeness by comparing socio-demographic characteristics of our study population to the ones of the FMH^39^ (age category, gender, and geographic region); see Supplementary Table 4.

### Survey administration

The self-administrated online survey was hosted on an institutional LimeSurvey server.^40^ A cookie-based prevention of multiple participation in the survey, regardless of the origin of the invitation, was enabled. At the end of the survey, a link was displayed to a separate form (not anonymised) to take part in a lottery with a chance of winning a Swiss Book Voucher with a value of 50 Swiss Francs (approximately GBP 46). Linking participants’ personal data with their survey answers was not possible.

### Survey dissemination

The survey link was disseminated in December 2023 via the Swiss Association of Family Medicine’s (MFE) e-mail newsletter (3825 members), as well as in January 2024 via the Swiss Society of General Internal Medicine’s (SSMIG) e-mail newsletter (8016 members). In order to increase the response rate, mailing lists of Sentinella epidemiologic surveillance network^41^ (181 members), teaching physicians of the Family Medicine Departments of Universities of Lausanne and Geneva (700 members), as well as University of Lucerne (344 members) were used as additional channels for dissemination. The survey was also promoted during a French-speaking continuous medical education (CME) event. Several social network posts from different academic and administrative institutions (Unisanté, 13 000 followers; Institute for Implementation Science in Healthcare, University of Zürich, 1000 followers; and the FOPH*,* Swiss Government, 110 000 followers) aimed to increase the visibility of the survey. The data collection ended on 29 February 2024.

### Ethical considerations

The survey was anonymous. Since the survey did not contain any identifying data nor patient information, ethical approval from ethics committee was not required according to the Swiss legislation.^42^ The study adhered to the principles of the Declaration of Helsinki, ensuring ethical considerations in the planning, execution, and reporting phases. During the research, the original dataset was stored in a Unisanté server (daily backups) with restrained security, accessible only to the project leaders (YM, SS). Data management was in conformation with Swiss legislation on data protection. All data was stored on Unisanté institutional repository.

### Data analysis

The data analysis was performed using RStudio for statistical computing.^43^ Questions with categorical or numerical answer modalities were mandatory and only questions with free-text answers were optional. Missing answers could occur in the case of drop-outs. They were not addressed or managed specifically.

### Quantitative analysis

We described proportions of responses for awareness, of the different AMS interventions targeting RTIs, and estimated mean scores with standard deviations for acceptability, appropriateness, and feasibility (Likert scale coded 1 to 5). Likert scales were dichotomised to positive opinion (‘I agree‘ and ‘I strongly agree‘) and negative opinion (‘I disagree‘ and ‘I strongly disagree‘). Neutral opinion (’I neither agree nor disagree’) were ignored in the dichotomised analysis. Association between the linguistic region and awareness of the interventions was evaluated using χ^2^ test. Association between linguistic region and the mean scores for acceptability, appropriateness, and feasibility was assessed using Welch’s two samples *t*-test. Each intervention’s acceptability, appropriateness, and feasibility were compared to the acceptability, appropriateness, and feasibility of all the other AMS interventions, using two-sample *t*-test. *P*<0.05 was considered statistically significant.

### Qualitative analysis

Inductive thematic analysis was employed to identify patterns and themes within the free-text answers of the survey.^44^ The authors considered the 2006 approach rather than Reflexive Thematic Analysis^45^ as appropriate in order to explore PCPs’ perspectives and expectations regarding an AMS intervention in primary care without focusing on interpretative nuances or the role of the researcher in theme development. Analysed answers included English-translated, free comments on each AMS intervention evaluated by PCPs targeting the antibiotic treatment of RTIs in primary care (survey part three), as well as the general expectations from PCPs regarding an AMS intervention efficiently supporting them in using antimicrobials (survey part five). The thematic analysis process began with data familiarisation, followed by the generation of initial codes to capture relevant concepts and ideas. Themes were identified, then reviewed between the three main authors through an iterative process of coding and categorisation, using Miro boards.^46^ The main themes and subthemes were finally named and refined, with consensus obtained between the three main authors.

## Results

### Characteristics of study sample

**Figure 1.**
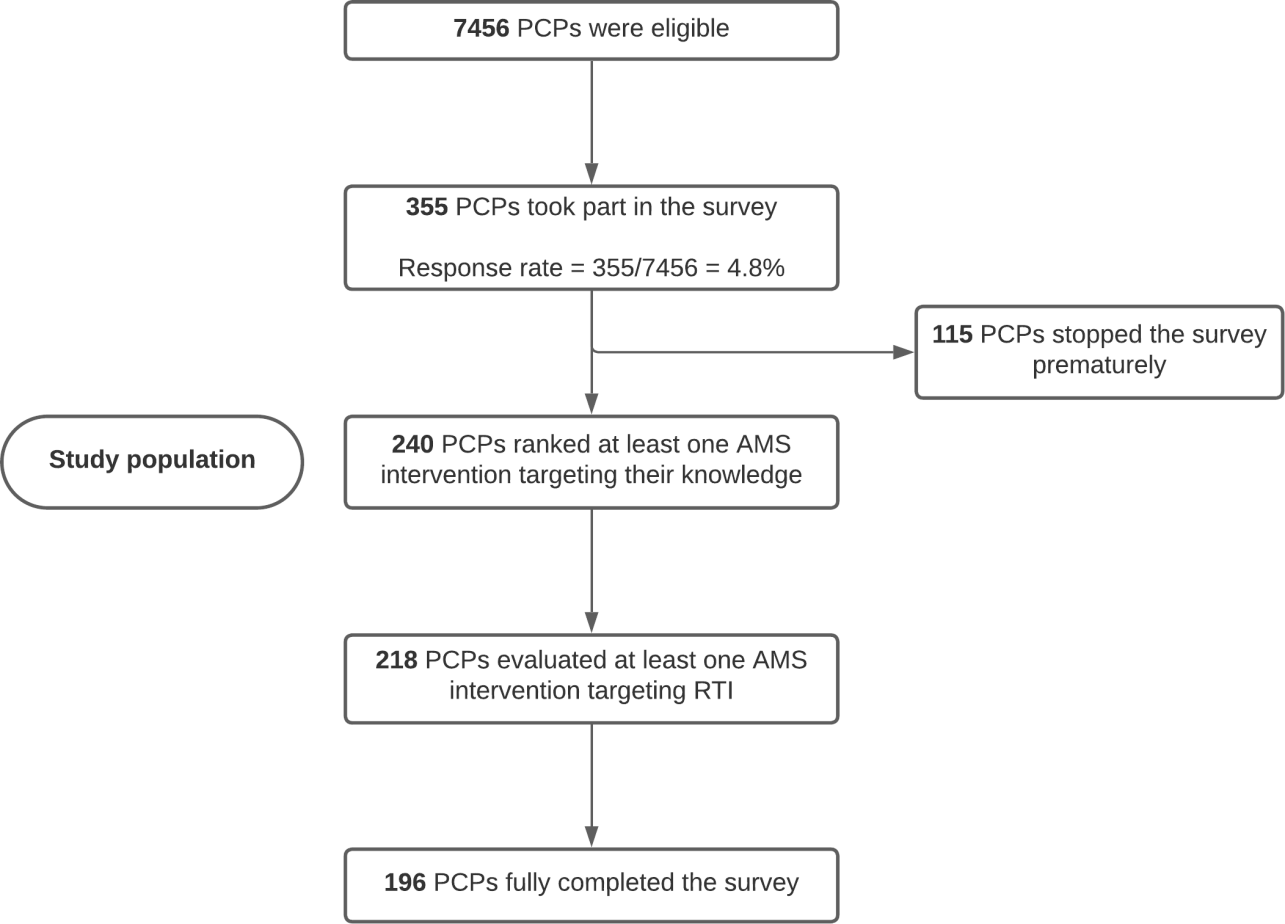
Flowchart of the survey evaluating antimicrobial stewardship interventions among Swiss primary care physicians

A total of 355 PCPs answered at least one question of the survey, representing 4.8% of all 7456 eligible physicians (see Figure 1). Of these, 240 PCPs ranked at least one AMS intervention targeting their knowledge (see Supplementary Table 3), and 218 PCPs evaluated acceptability, appropriateness, and feasibility of at least one intervention. In total, 196 PCPs fully completed the survey. As the main focus of the survey was the evaluation of the AMS intervention, the authors decided to consider the 240 that contributed to the ranking of the interventions as the study population. Out of the 240 participating PCPs, 108 (45.0%) were women, 121 (50.4%) were German-speaking, 113 (47.1%) were French-speaking, 228 (95.0%) were working in a private medical practice, and 225 (93.8%) were general internal medicine specialists (see Supplementary Table 3).

### Use of AMS interventions

Out of the 16 proposed sources of information about appropriate antibiotic prescription, the 240 PCPs stated referring primarily to regional hospital guidelines or nursing home guidelines (*n* = 172 ,71.7%), national guidelines (*n* = 160, 66.7%), education during congresses (*n* = 154, 64.2%), articles published in Swiss professional journals (*n* = 146, 60.8%), and infectious diseases specialist advice (*n* = 140, 58.3%), see Supplementary Table 5. The two least selected sources of information were the factsheets for physicians and WHO guidelines.

### Awareness of AMS interventions

PCPs were most aware of biomarkers to guide antibiotic prescription in RTIs (see Table 1), such as POC-PCT (67.6%) and POC-CRP (61.1%), and the FOPH awareness campaign (57.3%). The national guidelines website was familiar to 52.7% of PCPs. Awareness was lower regarding antibiotic prescription audit and feedback (36.0%), shared decision-making tools (27.0%), factsheets for physicians (22.5%), and communication skills training (17.1%). Among subgroups, German-speaking PCPs were more prone to know of the existence of the shared decision-making tools (36.2% versus 17.1%, *P* = 0.04).

**Table 1.** Primary care physicians’ awareness of antimicrobial stewardship interventions that can be used for respiratory tract infections in primary care

AMS intervention	Awareness Total, *n /N* ^b^ (%)	Awareness German-speaking, *n/N* (%)	Awareness French-speaking, *n/N* (%)	*P* value
**Use of PCT to guide antibiotic prescription**	69/102 (67.6)	28/49 (57.1)	38/50 (76.0)	0.05
**Use of CRP to guide antibiotic prescription**	58/95 (61.1)	30/51 (58.8)	26/40 (65.0)	0.55
**FOPH information material for patients**	47/82 (57.3)	28/42 (66.7)	19/38 (50.0)	0.13
Patient information leaflet	40/82 (48.8)	25/42 (59.5)	15/38 (39.5)	-
Poster	34/82 (41.5)	17/42 (40.5)	17/38 (44.7)	-
Educational video	3/82 (3.7)	1/42 (2.4)	2/38 (5.3)	-
Post-its	3/82 (3.7)	2/42 (4.8)	1/38 (2.6)	-
**National guidelines website (SSI)**	48/91 (52.7)	28/48 (58.3)	18/39 (46.2)	0.26
**Antibiotic prescription audit and feedback**	27/75 (36.0)	13/41 (31.7)	14/31 (45.2)	0.24
**Shared decision-making tools** ^a^	24/89 (27.0)	17/47 (36.2)	7/41 (17.1)	0.04
**Factsheets for medical doctors** ^a^	20/89 (22.5)	12/47 (25.5)	8/41 (19.5)	0.50
**Communication skills training program**	14/82 (17.1)	5/38 (13.2)	8/40 (20.0)	0.42

CRP = C-reactive protein. FOPH = Federal Office of Public Health. PCT = procalcitonin. SSI = Swiss Society of Infectious Diseases.

^a^Awareness was assessed separately for the factsheets and the shared decision-making tools, but each time by the same PCPs, as those interventions were meant to be used together. ^b^N varies across intervention, as 2 AMS interventions and 1 biomarker were randomly attributed to each participating PCPs.

### Acceptability, appropriateness, and feasibility of AMS interventions

**Figure 2.**
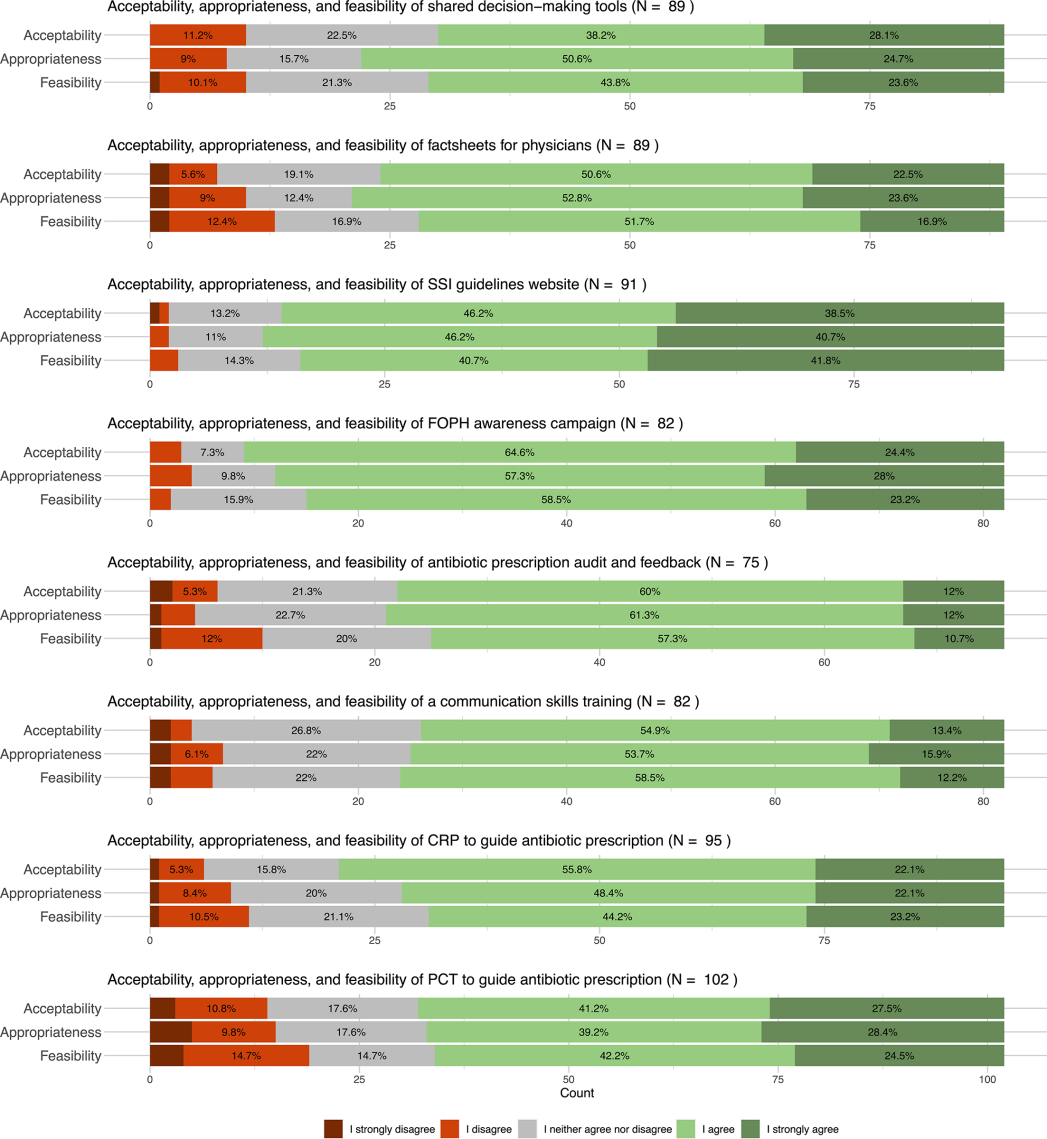
Acceptability, appropriateness, and feasibility of antimicrobial stewardship interventions targeting respiratory tract infections to Swiss primary care physicians, as proportions of a 5-point Likert scale (proportions not displayed if <5%). CRP = C-reactive protein. FOPH = Federal Office of Public Health. PCT = procalcitonin. SSI = Swiss Society of Infectious Diseases.

All AMS interventions were rated as acceptable (mean acceptability out of five = 3.89, SD = 0.17), appropriate (mean appropriateness out of five = 3.91, SD = 0.18), and feasible (mean feasibility out of five = 3.81, SD = 0.20) by participating PCPs. The national guidelines website and the FOPH information material were evaluated as the most acceptable, appropriate, and feasible interventions, achieving the most positive opinions and the least negative opinions (see Figure 2). The national guidelines website attained mean scores for acceptability, appropriateness, and feasibility 4.20, 4.25, and 4.21 out of 5, respectively (*P*<0.001 versus the scores of the other interventions, see Supplementary Table 6). The FOPH information material obtained mean scores for acceptability, appropriateness, and feasibility of 4.10, 4.09, and 4.02 out of 5, respectively (*P* = 0.01, 0.03 and 0.01, respectively). In contrast, antibiotic prescription audit and feedback was evaluated as less acceptable, appropriate, and feasible than the other interventions, with mean scores of 3.72, 3.78, and 3.63 out of 5, respectively (*P* = 0.07, 0.12, and 0.06, respectively). Although the acceptability, appropriateness, and feasibility scores of POC-PCT were among the lowest overall (with mean scores out of 5 of 3.76, 3.79, and 3.69, respectively, also obtaining the most negative opinions, see Figure 2), mean scores were significantly higher among French-speaking PCPs than among German-speaking PCPs (acceptability of 4.14 vs 3.47, *P* = 0.002; appropriateness of 4.04 versus 3.61, *P* = 0.030; and feasibility of 4.06 versus 3.41, *P* = 0.002). The acceptability, appropriateness, and feasibility of a communication skills training program was also rated significantly higher among French-speaking PCPs than among German-speaking PCPs (acceptability of 4.00 versus 3.50, *P* = 0.003, appropriateness of 3.95 versus 3.53, *P* = 0.020, and feasibility of 3.90 versus 3.53, *P* = 0.030).

### Perceived time required to perform AMS interventions

Median physicians’ estimation of the time needed to interpret one quarterly report was 15 minutes per report (see Supplementary Table 7). Other interventions had a median estimated time added per consultation from 0(use of PCT to guide antibiotic prescription) to 5 minutes (shared decision-making tool).

### PCP preferences regarding antibiotic prescription audit and feedback

The Swiss Center for Antibiotic Resistance (anresis.ch) was the most accepted source of feedback regarding a national antibiotic prescription audit and feedback intervention, 58.7% of answers, followed by quality circles (40.5%) and an academic institution (40.5%, see Supplementary Table 8). PCPs also expressed their preference for being compared to physicians of the same specialty (either in their canton, 72.0%, or nationally, 64.0%), and to receive a quarterly report (68.0%).

### PCPs’ expectations about an AMS intervention in Switzerland

#### Main themes

**Figure 3.**
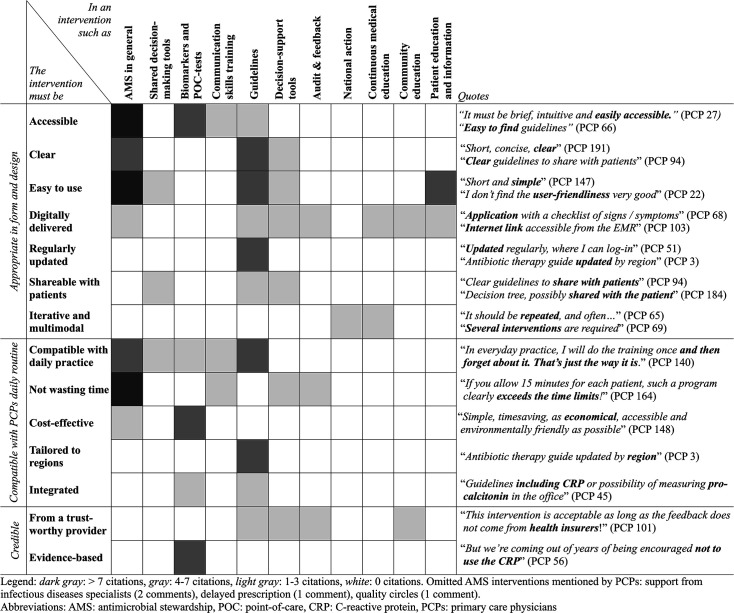
Primary care physicians’ expectations (main attributes) regarding antimicrobial stewardship interventions

Inductive thematic analysis of 151 free-text answers regarding PCP’s expectations about an AMS intervention identified three main themes: appropriateness of form and design of the intervention; compatibility of the intervention with PCPs’ daily routines; and credibility of the source of the intervention (see Figure 3)

#### Interventions that are appropriate in their form and/or design

Accessibility of AMS interventions resonated strongly among PCPs, with many expressing a desire for *’easily accessible resources*’, and interventions that are *’quickly available*’, and free to access (*’offering PCPs literature about antibiotics for free!*’). Those easy-to-access information sources included guidelines (’*easy-to-find guidelines*’), infectious diseases specialist’s advice (*’easy access to a local infectiologist*’), or point-of-care tests (*’PCT available at the office*’). Straightforwardness of the intervention was also emphasised by PCPs, who brought out the importance of interventions being *’clearly structured*’and providing *’clear information*’, especially regarding guidelines, which must be *’clear statements*’, or decision-aid algorithms. Additionally, the ease of use was also identified as a key attribute desired in AMS in general, with PCPs emphasising the need for interventions to be *’easy to understand*’ for both the physician and the patient. For instance, patient-education material was often evaluated as containing *’too much text*’, or to be lacking *’iconography*’. Finally, digitally delivered interventions emerged as a significant aspect of PCPs’ expectations. PCPs expressed interest in embedded features, such as an *’internet link accessible from the electronic medical record*’and suggested that *’online training* […] *would be a good idea*’. Digital aspects were mentioned in a broad range of interventions, such as an online guidelines repository (*’A comprehensive online guideline, updated annually by* [a professional organisation]*, like Sanford,* ^47^ *but for Switzerland*’), community education (*’federal campaign through all channels’*), patient education (*’serious games*’), antibiotic prescription audit and feedback (*’brief, informative e-mails from loco-regional reference organisations*’), and decision-support tools (*’smartphone application with a checklist of signs and symptoms*’). Some PCPs also insisted on the multimodal aspects of AMS in general: *’up-to-date guidelines, magazine, article, training, several interventions are required*’.

#### Interventions that are compatible with daily practice

Compatibility with daily practice was noted as essential by PCPs, who emphasised the importance of interventions being *’relevant to practice*’ and *’practical*’. Perceived incompatibility with daily practice was seen in several evaluated interventions, such as the national guidelines website (*’recommendations sometimes not at all adapted to clinical reality*’), communication skills training (*’In everyday practice, I will do the training once and then forget about it. That’s just the way it is*.’), point-of-care tests, or shared decision-making tool. PCPs also indicated a preference for interventions that are *’brief*’ and ‘*short*’. This was especially notable when evaluating the communication skills training program (*’If you allow 15 minutes for each patient, such a program clearly exceeds the time limit!*’) or decision-support tools (judged as *’too long*’).

#### Credibility of source of the intervention

Finally, PCPs expressed enthusiasm in interventions developed by trustworthy institutions, such as decision-support tools originating *’from the reference cent*re’, *’independent*’ guidelines, or a *’federal*’ awareness campaign. This sentiment was particularly pronounced when evaluating antibiotic prescription audit and feedback. Notably, PCPs displayed a clear aversion to receiving feedback from insurance companies: *’This intervention is acceptable as long as the feedback does not come from health insurers!*’. Finally, PCPs emphasised the importance of evidence-based interventions, particularly concerning the use of biomarkers: *’I’ve been doing it for a long time, I can’t say whether this saves anything*’.

## Discussion

### Summary

The main objective of this study was to evaluate the present use of, awareness about, and perceived acceptability, appropriateness, and feasibility of a broad range of AMS interventions available for Swiss PCPs. Overall, all interventions evaluated by PCPs had good acceptability, appropriateness, and feasibility (>3.6 out of 5). However, apart from biomarkers that were familiar to most PCPs, many interventions developed in the Swiss context did not reach their target population. Our results show that many PCPs are not aware of interventions that are rated as acceptable, appropriate, and feasible.

### Strengths and limitations of the study

As study participants were self-selected, PCPs taking part in this study are susceptible to have a more positive view towards AMS in Switzerland, which might overestimate the awareness, acceptability, appropriateness, and feasibility of those interventions. By disseminating our survey via teaching PCPs’ mailing list, our sampling strategy favoured the inclusion of PCPs involved in teaching to medical students, who may be more aware of AMS interventions than other PCPs. Also, our study sample tends to over-represent younger and French-speaking PCPs (see Supplementary Table 4). While PCPs who had previously participated in AMS trials were included in the study, potentially leading to an overestimation of awareness, acceptability, appropriateness, and feasibility of certain AMS interventions, excluding them — particularly in the audit and feedback trial with over 3000 participants^17^ —would have significantly impacted the representativeness of our findings. Finally, desirability bias is possible in such a survey, affecting mostly awareness results. Nevertheless, this study underscores the importance of initiatives aimed at promoting AMS among PCPs, while elucidating their preferences and viewpoints regarding a broad range of interventions.

### Comparison with existing literature

This study was the first nationwide survey assessing physicians’ perspectives of a broad range of AMS interventions. The response rate of our study is difficult to estimate precisely, since our survey was promoted via several channels, and the number of physicians having seen the survey link is impossible to determine. However, our study managed to attain a higher number of responding PCPs than a similar survey evaluating Swiss PCPs’ perspectives on POC-CRP in the management of RTIs, in which 188 PCPs answered at least one question.^48^ Although evaluated as being the most acceptable, appropriate, and feasible intervention, barely half of responders were aware of the national guidelines’ website. To the author’s knowledge, no study reported the awareness levels of antibiotic prescription guidelines among PCPs, although some hospital-based surveys reported awareness rates from 75% to 98%.^49,50^ Nevertheless, with only 53% of PCPs being aware of the national guidelines’ website, the promotion of this tool could be worthwhile. Participating PCPs had a preference in using local or regional guidelines or reading Swiss scientific articles rather than international ones. AMS interventions could therefore be adapted to the national or regional needs in terms of antibiotic resistance. This finding could also reflect increased trust in local or regional experts. The present study also sheds light on PCPs’ preferences regarding the design of an antibiotic prescription audit and feedback intervention within the Swiss healthcare landscape, as some studies concluded that stakeholders’ perspectives on audit and feedback highly depend on the form and design of such an intervention.^51,52^ Regarding biomarkers to guide antibiotic prescription in RTIs, the use of PCT had divergent results between the two main linguistic regions of Switzerland, with French-speaking PCPs showing significantly higher ratings. This is probably attributable to the UltraPro trial that aimed to evaluate the use of procalcitonin in the management of RTIs in primary care, which took place in the French-speaking part of Switzerland.^14^ A qualitative study on the acceptability of POC-PCT showed mostly positive attitudes with an enhancement of self-confidence, high trust in the results, and improved patient-physician relationship.^53^

### Implications for practice

Qualitative analysis of PCPs’ free-text comments provided valuable viewpoints on AMS interventions, emphasising the importance of form, design, compatibility with daily practice, and the trustworthiness of their source. Feasibility ratings were often judged more negatively than acceptability or appropriateness for several interventions. This highlights the challenges of integrating interventions with daily practice (for example, a biomarker perceived as costly or complicated to use, a factsheet or quarterly feedback perceived as time-consuming). Interestingly, the feasibility of using POC-PCT was judged significantly higher among French-speaking PCPs, some of whom were already familiar with POC-PCT because of the UltraPro trial, compared to German-speaking PCPs. This highlights the importance of being able to try out a specific intervention to overcome some barriers to implementation.

### Implications for research

Even though this study evaluated three implementation outcomes of AMS interventions, these outcomes are not the only ones being worth extensive investigation, such as adoption, costs, or penetration. Further research could dig deeper into PCP perspectives, attitudes, and views of the different AMS interventions, to design AMS interventions, ultimately increasing their uptake. Finally, the involvement of PCPs in the evaluation, design, and promotion of AMS interventions is essential to gain a comprehensive understanding of their needs and preferences, thus facilitating the development and uptake of effective interventions, ultimately contributing significantly to the collective efforts against antimicrobial resistance and public health protection.

## Data

Supplementary Table 1: Antimicrobial stewardship interventions potentially influencing primary care physicians’ knowledge on appropriate antibiotic prescription (survey part II). Supplementary Table 2: Antimicrobial stewardship interventions targeting the antibiotic treatment of respiratory tract infections evaluated by Swiss primary care physicians (survey part III). Supplementary Table 3: Comparison of characteristics of study sample with characteristics of specialists in general internal medicine and practicing physicians working in outpatient sector according to Swiss Medical Association statistics. Supplementary Table 4: Comparison of characteristics of study sample with characteristics of specialists in general internal medicine and practicing physicians working in outpatient sector according to Swiss Medical Association statistics. Supplementary Table 5: Top 5 most used antimicrobial stewardship interventions by primary care physicians when looking for information, or to update their knowledge about appropriate antibiotic prescription, total and by language of response. Supplementary Table 6: Mean scores for acceptability, appropriateness and feasibility of antimicrobial stewardship interventions that can be used for respiratory tract infections in primary care, overall and among French, and German-speaking primary care physicians, on a five-point Likert scale. Supplementary Table 7: Primary care physicians’ estimated time to use antimicrobial stewardship interventions targeting antibiotic treatment of respiratory tract infections. Supplementary Table 8: Primary care physicians’ preferences regarding contextual factors of antibiotic prescriptions audit and feedback interventions. SupplementaryDocument.docx: full length questionnaire.

## Data availability

Anonymised dataset (full dataset except free-text items), metadata, and documentation (full length questionnaire, data dictionary) are available for replication and research purpose on Unisanté Data Repository under the license CC-BY for at least 10 years (until 2034) on : https://doi.org/10.16909/dataset/49.
